# EagleEye: A Worldwide Disease-Related Topic Extraction System Using a Deep Learning Based Ranking Algorithm and Internet-Sourced Data

**DOI:** 10.3390/s21144665

**Published:** 2021-07-07

**Authors:** Beakcheol Jang, Myeonghwi Kim, Inhwan Kim, Jong Wook Kim

**Affiliations:** 1 Graduate School of Information, Yonsei Univeristy, Seoul 03722, Korea; bjang@yonsei.ac.kr (B.J.); moreih29@gmail.com (I.K.); 2 Department of Computer Science, Sangmyung University, Seoul 03016, Korea; nopkgogo3@gmail.com

**Keywords:** internet-sourced data, disease-related topic ranking, Word2Vec, BiLSTM, TF-IDF, WBiLSTM-TF-IDF

## Abstract

Due to the prevalence of globalization and the surge in people’s traffic, diseases are spreading more rapidly than ever and the risks of sporadic contamination are becoming higher than before. Disease warnings continue to rely on censored data, but these warning systems have failed to cope with the speed of disease proliferation. Due to the risks associated with the problem, there have been many studies on disease outbreak surveillance systems, but existing systems have limitations in monitoring disease-related topics and internationalization. With the advent of online news, social media and search engines, social and web data contain rich unexplored data that can be leveraged to provide accurate, timely disease activities and risks. In this study, we develop an infectious disease surveillance system for extracting information related to emerging diseases from a variety of Internet-sourced data. We also propose an effective deep learning-based data filtering and ranking algorithm. This system provides nation-specific disease outbreak information, disease-related topic ranking, a number of reports per district and disease through various visualization techniques such as a map, graph, chart, correlation and coefficient, and word cloud. Our system provides an automated web-based service, and it is free for all users and live in operation.

## 1. Introduction

Humans have suffered from various infectious diseases such as COVID-19, SARS, Ebola, and MERS. With increased traffic, coupled with effective communication and globalization, there is an unprecedented rapid spread of diseases all over the world, and their damages are increasing. Even with the remarkable development of medicine and significant leaps in vaccine development, it is becoming almost impossible to treat all newly detected diseases, and the intuitive solution seems to be the prevention and containment of epidemic diseases through effective earlier warnings. Such prevention can be achieved by leveraging all available data, not only structured and formal but also unstructured and noisy, to build robust systems that can help prevent the spread of diseases through accurate monitoring and public awareness. Since the end of World War II, many countries have established their own Centers for Disease Control and Prevention (CDCs), main goal of which the is to protect public health through the control and prevention of disease. CDCs surveil disease outbreaks, but they require a lead time of more than one week to collect and produce disease outbreak statistics, which makes it difficult to respond instantly to new disease outbreaks.

Internet-sourced data such as online news (i.e., Microsoft Bing News [1]), Social Networks Services (SNS) (i.e., tweets [2]) and web search queries (i.e., Google Trends [3]) that contain information entered by the public have quickly increased in terms of size and coverage and have shown non-substitutable potential to detect disease outbreaks much earlier [4,5]. Internet-sourced information on disease outbreaks can be recorded and spread on the Internet much faster than CDCs’ announcements. However, such information is usually hidden in extremely noisy, sparse, unstructured and untrustworthy websites and social networks, making them hardly discoverable and barely trustable once discovered [6]. Previous studies have developed real-time disease surveillance systems by analyzing the correlation between the frequency of disease outbreak and the frequency of related Internet-sourced data. However, existing real-time disease monitoring systems are designed only for a specific region or specific disease. In addition, the keyword-based data collection method to obtain the frequency of related Internet source data does not handle noise in the data. Therefore, there is a need for a worldwide disease monitoring system that filters out noisy or unreliable data and performs real-time monitoring for multiple diseases and regions.

In this paper, we present a novel worldwide disease-related topic extraction system called EagleEye. To do that, we propose and implement an effective deep-learning-based ranking algorithm referred to as WBiLSTM-TF-IDF, which extracts a list of important words from Internet-sourced data. The approach leverages Word2Vec [7], BiLSTM [8] and Term Frequency-Inverse Document Frequency (TF-IDF) [9] algorithms. Word2Vec [10] is used to obtain word embeddings, a distributed word representation that expresses the semantic similarities between words. BiLSTM uses vectors for a binary classification that determines the relatedness of the data to disease outbreaks. TF-IDF extracts and ranks disease-related hot words using only disease-related data classified by BiLSTM. EagleEye exploits a variety of Internet-sourced data such as online news articles, SNS and web search query. With this system, the users can view the current as well as past records. In addition, it provides a user-friendly interface exploiting a variety of effective visualization techniques such as a table, graphs, correlation coefficient and a word cloud.

EagleEye presents the following disease-related information to users. Firstly, EagleEye collects disease-related online news and SNS data and classifies them by their nations and districts. It presents a list of disease-related hot words together with corresponding news articles and tweets. It also provides valuable disease statistics such as the top five districts with the selected disease, the top five diseases, the monthly top disease and the word cloud of news articles and tweets. Secondly, EagleEye provides useful statistics on Google search queries by the selected nation and disease such as the weekly amount of disease-related Google search queries and the top five districts with these disease-related Google search queries. Thirdly, EagleEye presents useful statistical comparison results of collected Internet-sourced data such as numbers by time, their correlation coefficien, and the top five diseases observed in a selected nation.

Finally, we conducted deep experiments to evaluate the performance of our proposed ranking algorithm and system. Experimental results show that our ranking algorithm is more accurate and better detects disease-related events than existing ranking algorithms and that our system is more sensitive to disease-related events and collects more relevant data than conventional systems. As per our knowledge, only a few related systems are in operation. EagleEye is a fully developed automated web-based disease outbreak surveillance system, has been in operation since January 2019 and is free for all users. The system is currently available online at http://www.epidemic.co.kr/worldwide/news (accessed on 6 July 2021), http://www.epidemic.co.kr/worldwide/twitter (accessed on 6 July 2021), http://www.epidemic.co.kr/worldwide/trend (accessed on 6 July 2021), and http://www.epidemic.co.kr/worldwide/comparison (accessed on 6 July 2021).

## 2. Related Work

Recently, the use of Internet-sourced data for public health and disease surveillance has received a great deal of attention. This section examines systems and related research that leveraged Internet-sourced data focusing on online news articles, SNSs and Google search queries for disease tracking.

HealthMap [11] is a system that collects user’s reports as well as Internet news articles from around the world and displays disease information to the user through a map. In HealthMap, the collected data are classified into existing locations and disease patterns using the N-gram approach and stored in a database. It consists of 2300 places and 1100 disease variants. The data stored in the database are displayed on an interactive map for easy access. EpiSPIDER [12] is another system that collects data from Promed [13] and the Global Disaster Alert and Coordination System (GDACS) and uses askMEDLINE to link PubMED articles with information extracted from Promed. Automated processes are used to display data on maps via Yahoo and Google map API, showing histories of each disease over three years on the bar graph. GPHIN [14] is an early disease warning system that monitors global media sources, collects and disseminates information about disease outbreaks and other public health events to alert international organizations promptly. BioCaster [15] is an ontology-based text mining system for detecting and tracking infectious disease outbreaks from Internet-sourced data by continually analyzing the documents reported in the RSS feed and categorizing geographic relevance and displaying them on Google maps. It collects Internet-sourced data on an hourly basis through an RSS feed, and the collected data are categorized by disease name and geographical location through BioCaster ontology.

Social media contain rich unexplored information about people’s personal health, symptoms of illness, treatments and adverse drug events because people may choose to disclose their health issues in social acquaintances rather than formal hospital settings. Among them, Twitter is proposed as a disease surveillance data source because it has the potential to generate millions of posts per day and provide real-time access. Researchers at Iowa University [16] used content from the Twitter stream to track rapidly changing public sentiment about H1N1 influenza and swine flu as well as to measure the actual disease activity. Since 29 April 2009, these researchers have created a classification system by collecting tweets that match predefined search terms such as influenza, swine and vaccines. The classification system constructed an automatic supervised learning method using Support Vector Regression, which is an example of SVM (Support Vector Machines). It was also used to compare influenza-like illness (ILI) data from the Centers for Disease Control and Prevention (CDC) and Influenza Sentinel Provider Surveillance Network data to compare with real-world cases. David A. Broniatowski et al. [17] described a system for monitoring influenza using social media data. The biggest disadvantage of social media data is that they contain many casual terms that are difficult to discern using analytical solutions; hence, the study used a new infection detection algorithm to build a binary classification model that successfully filters unrelated tweets. The actual data and results of influenza from the CDC showed that the system detected 85% of the influenza epidemic shifts and doubled the accuracy compared to the simple model. It also predicts that CDC data will be forecast on a weekly basis. Northwestern University researchers [18] used Twitter data to build a real-time surveillance system for cancer activity. The proposed system uses Twitter and stream APIs to store cancer-related data and then applies space, time and text models to discover topics related to national flu and cancer activity and disease-related terms. The system consists of clusters of transaction databases as well as high-dimensional data warehouses that are updated and viewed in real time through pie charts, time series graphs and US disease activity maps. The goal of the study is to track the spread of the disease in the United States by measuring the amount of cancer-related data generated in the country. The proposed disease detection system shows that the level of cancer activity in the United States can be mapped closer to real time, hence facilitating the visualization and comparison of cancer related terms, cancer types, symptoms and treatment in terms of their popularities.

As billions of people resort to online search for health-related information and as their searches span a wide range of subjects, web search queries become a useful source of information, and Google Trends is a market leader in this endeavor that has attracted a lot of research aiming at disease surveillance and tracking. Johns Hopkins University team [19] developed the GARMA (Generalized Autoregressive Moving Average) model with discrete value distribution and integrated external variables for anticipatory alerting of influenza cases to the medical center and real-time influenza surveillance. The proposed predictive model yields predictions by integrating climate with key causal variables (former influenza cases) with some external variables related to influenza surveillance (influenza virus type, number of emergency medical services calls, sick leave and counter drug sales). As a result, the predictive model predicted 83% of cases of infection and was practically useful by providing a warning of influenza outbreaks. Min Kang et al. [20] compared the influenza data collected by Guangdong CDC (GCDC) with Google trends from 2008 to 2011 and proved that Google Trends can be used as a complementary data source for influenza surveillance in southern China. In the study, Pearson correlation coefficients with a 95% confidence interval were calculated for all data and each year to compare GCDC data with Google Trends data. The highest correlation was 0.73 (95% CI: 0.66, 0.79) between Google Trends and influenza monitoring for FEVER, proving the usefulness of Google Trends data for influenza surveillance. JianYing Wang et al. [21] conducted research using Google Trends data to predict outbreaks of bovine stomatitis. To analyze the occurrence of bovine stomatitis in the United States in 2014, these researchers collected data on the occurrence of bovine stomatitis from the World Organization for Animal Health (OIE) and collected bovine stomatitis keyword data through Google Trends. They calculated the correlation of Pearson and Spearman coefficients, and the results showed that there is a relationship between the keywords derived from Google Trends and the occurrence of bovine stomatitis. The researchers also constructed a disease prediction model using two types of methods. The predictive model constructed a qualitative classification model and the quantitative regression model using Google Trends data to predict the outbreak of the bovine stomatitis with a high accuracy, hence effectively containing the disease risks. The researchers concluded that early-warning systems for bovine stomatitis were an important area of study in some countries, and effective early-warning systems could significantly reduce livestock losses. Finally, YueTeng et al. [22] developed a dynamic prediction model for the Zika virus based on real-time online search data from Google Trends. In the study, the researchers confirmed a strong correlation between the Zika-virus-related Google Trends data and the cumulative numbers of reported cases and developed an automatic regression integrated moving average (ARIMA) model for dynamic estimation of Zika virus outbreaks. The researchers used Pearson Product–Moment Correlation to evaluate the linear correlation of Google Trends data related to the Zika virus and experimentally showed that the predicted data by the ARIMA model are remarkably similar to the real data of the Zika virus.

In this paper, we build an automated web-based disease outbreak monitoring system that overcomes the limitations of the existing disease-related studies described above and applying a deep-learning-based model. Our main contributions are as follows: collection of various infectious disease-related Internet-sourced data, deep-learning-based data filtering and ranking algorithm for important disease-related topic extraction, and a user-friendly and convenient web system.

## 3. Method

**Figure 1:**
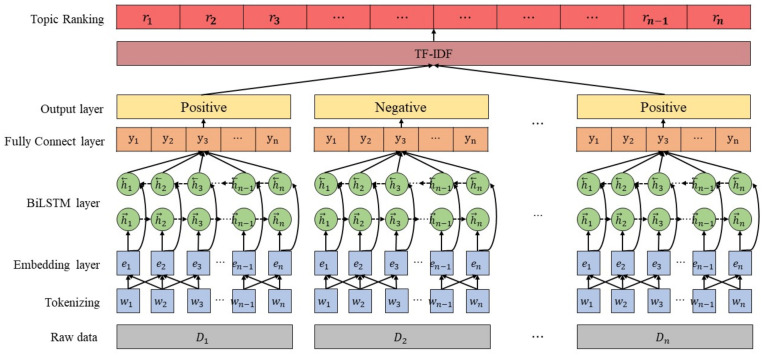
Model architecture of our proposed deep-learning-based ranking algorithm, WBiLSTM-TF-IDF.

This section describes our proposed data filtering and ranking algorithm, WBiLSTM-TF-IDF, including its data acquisition method. The model filters out the data not related to disease outbreak using Word2Vec and BiLSTM and ranks disease-related hot words using TF-IDF. Figure 1 presents the overall architecture of WBiLSTM-TF-IDF.

WBiLSTM -TF-IDF filters out news articles and tweets not related to disease outbreaks by combining Word2Vec and BiLSTM. Word2Vec is one of the most effective word embedding technologies and improves the performance of natural language processing by vectorizing words considering the meaning of words [23]. BiLSTM is a neural network model that extracts features from both directions of a sentence and shows superior performance compared to other neural network models in sentence classification [24]. Raw data such as news articles and tweets are tokenized into individual words ($w_{1}\ldots w_{n}$). The embedding layer utilizes Word2Vec’s Skip-gram [25,26] architecture to obtain ($e_{1}\ldots e_{n}$) a distributed representation of obtained tokens. The Skip-gram architecture helps the model to express words as per their semantic similarities by adjusting the probabilities of the proximity words that appear in context with a given word. Given a sentence consisting of words ($w_{1}\ldots w_{n}$), the following equation is used to learn the corresponding vector ($e_{1}\ldots e_{n}$).

(1) $$\frac{1}{T}\sum_{t=1}^{T}\sum_{-c\leq j\leq c,j\neq 0}\log p\left(w_{t+j}|w_{t}\right)$$

The vector values obtained from the embedding layer are fed into the BiLSTM layer. BiLSTM [27] is a sequential learning model used to learn input values according to time and order. The resulting values ($y_{1}\ldots y_{n}$) are fed into the fully connected layer after the calculation of the forward layer and the backward layer using the following equations [28].

(2) $$\vec{h_{n}}=H\left(W_{e\vec{h}}e_{n}+W_{\vec{h}\vec{h}}h_{n-1}+b_{\vec{h}}\right)$$

(3) $$\overset{\leftarrow }{h_{n}}=H\left(W_{e\overset{\leftarrow }{h}}e_{n}+W_{\overset{\leftarrow }{h}\overset{\leftarrow }{h}}h_{n-1}+b_{\overset{\leftarrow }{h}}\right)$$

(4) $$y_{n}=W_{\vec{h}y}{\vec{h}}_{n}+W_{\overset{\leftarrow }{h}y}{\overset{\leftarrow }{h}}_{n}+b_{y}$$

The values that are fed into the fully connected layer ($y_{1}\ldots y_{n}$) are classified into positive and negative classes using the SoftMax probability function. Eventually, WBiLSTM-TF-IDF extracts only the news or tweets that are related to disease outbreaks.

WBiLSTM-TF-IDF extracts the ranking of important disease-related topics in real time from news articles and tweets obtained through the data acquisition process. It uses the ranking algorithm to extract important disease-related topics. Ranking algorithms were used in the early IR field to find words that were frequently mentioned in the document. WBiLSTM-TF-IDF uses the TF-IDF algorithm as a ranking algorithm. The TF-IDF algorithm is as follows.

(5) $$TF-IDF=tf\left(t,d\right)\times \log\frac{D}{1+df(t)}$$

In Equation (5), tf(t,d) means the frequency of how many times the word *t* appears in sentence *d*. $\log\frac{D}{1+df(t)}$ is the value obtained by dividing the total number of sentences by the number of sentences in which the word appears and indicates how commonly a word appears in the entire document [29].

To provide real-time disease-related information, our proposed system, EagleEye, collects and exploits three kinds of Internet-sourced data; news articles, Social Network Services (SNSs) and web search queries. Firstly, EagleEye collects online news articles using Microsoft Azure news search API [1] by sending search queries to Microsoft Bing News and gets a list of related news articles. Bing News is one of the most powerful news aggregators in the world and is suitable for gathering worldwide disease-related news articles because it processes billions of global searches and provides category-specific articles as well as the latest news from all around the world. Secondly, EagleEye collects tweets from Twitter [30] to acquire SNS data. Twitter is one of the biggest and most popular SNS providers and has attracted the interest of top research that aims to leverage this potential to detect disease-related events [31]. Our system collects online news articles and tweets using disease-related keywords such as disease names and symptoms from twenty major countries distributed across all seven continents. EagleEye includes the list of dangerous infectious diseases that are confirmed and made public by the World Health Organization (WHO) and the United Nations (UN), as well as the list of associated symptoms that characterize these diseases. To obtain disease-related data of important districts, it also collects disease-related data of districts where international airports are in the country to collect local data for each country. The international airport is one of the busiest areas in the country that attracts locals and foreigners who are hence vulnerable to an increased risk of diseases proliferation. Because the regional divisions of each country are quite different, we choose the districts with international airports as areas of interest. Finally, we use Google Trends to acquire data from web search queries. People search for disease-related information using a variety of web portals. Google is a major portal site, and its search query data can be exploited to identify trends in the disease, depending on how the disease is being searched. To do this, we collected search query data from Google Trends [32], which provides search query statistics based on queried keywords.

### System Architecture

**Figure 2:**
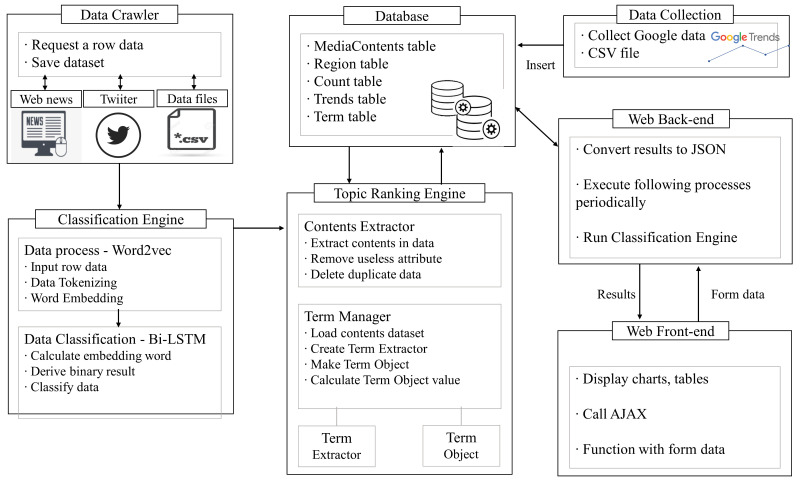
System architecture of our proposed system.

This section presents the details of the modules that make up our system. Figure 2 presents the system architecture of EagleEye. The data crawler collects news and SNS data by collecting Bing News articles using Azure API and tweets using Twitter API. The APIs [33] send the URL in JSON [34] format through HTTP [35] GET request. The collected data are stored in a CSV file with the search keyword, date, continent and selected country. The collected data files are sent to the classification engine. The data crawler gathers search query statistical data from Google Trends, which are stored in the database in the form of a CSV file. Data from Google Trends are collected using Pytrends API [36]. The classification engine consists of Word2Vec and Bi-LSTM. Using Word2Vec [37], each word is expressed by a vector value that represents the semantic closeness, and BiLSTM determines if the data are related to disease outbreaks. As a result, the classification engine extracts only the data related to disease outbreaks and sends them to the topic ranking engine.

The topic ranking engine consists of the content extractor and the term manager. The content extractor checks duplicate and sentence similarity and eliminates unnecessary elements (URL, html, tags, etc.) from the data using Sift4 [38], and then the refined data are sent to the MediaContents_tbl table of the database. The term manager includes the term extractor and the term object. The term extractor loads the data stored in MediaContents_tbl to generalize and tokenize the data. The generalization eliminates misspellings and words that do not match the grammar, and the tokenization collects only nouns. Our system uses the Natural Language Toolkit (NLTK) [39], a Python package used for the tokenization and generalization and developed for research and education. The generalized and tokenized data form a token list to constitute a term object. As a result, the collected data are stored in the term object list after unnecessary elements and meaningless words are removed, and the disease topic keyword ranking is generated by using the TF-IDF algorithm.

The web back-end returns the data requested from the web front-end to JSON type and runs the classification engine module according to the job scheduler time. We used Spring4 [40], Jetty [41] and Model View Controller (MVC) [42] to build the web back-end of our proposed system.

The web front-end provides the user interface for data acquisition and visualization. It requests information using jQuery [43] and data with the server using JavaScript and XML (AJAX) [44]. The data received from the server is in JSON format, and the web front-end uses them to generate maps, chart graphs and word clouds. In our proposed system, the dataTable.js [45] library is used to generate a table of disease keyword rankings, D3.js [46] is used to create a word cloud, and chart.js [47] library is used to create charts and graphs exchanges data with the server using JavaScript and XML (AJAX).

## 4. Results

Our system provides four disease-related web interfaces. The first interface presents data based on Bing news, the second interface displays data based on tweets, the third interface provides data based on Google search, and the fourth interface does a comparison of the three types of data. In this section, we explain the results of each page thoroughly.

**Figure 3:**
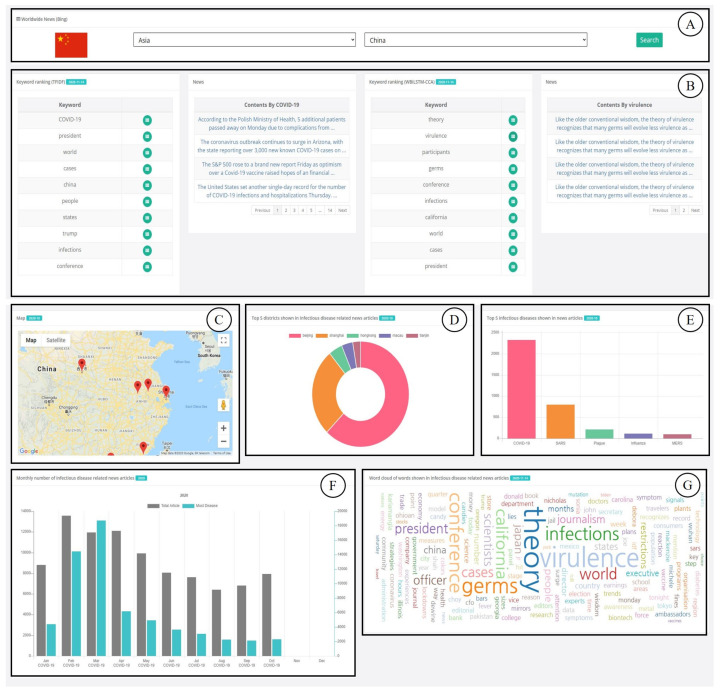
Web interface based on Bing News articles: (**A**): input form that allows the user to select the continent and country. (**B**) disease-related topic ranking based on the news articles collected with the disease keyword. (**C**) a map of the country (**D**) the raking of the top five districts where the largest number of disease-related news articles is reported. (**E**) the ranking of the top five diseases most frequently mentioned in disease-related news articles of the selected nation. (**F**) the trend of this year for a monthly total number of collected news articles and a monthly number of news articles of the most frequently shown disease. (**G**) disease-related word cloud based on collected disease-related news articles.

**Figure 4:**
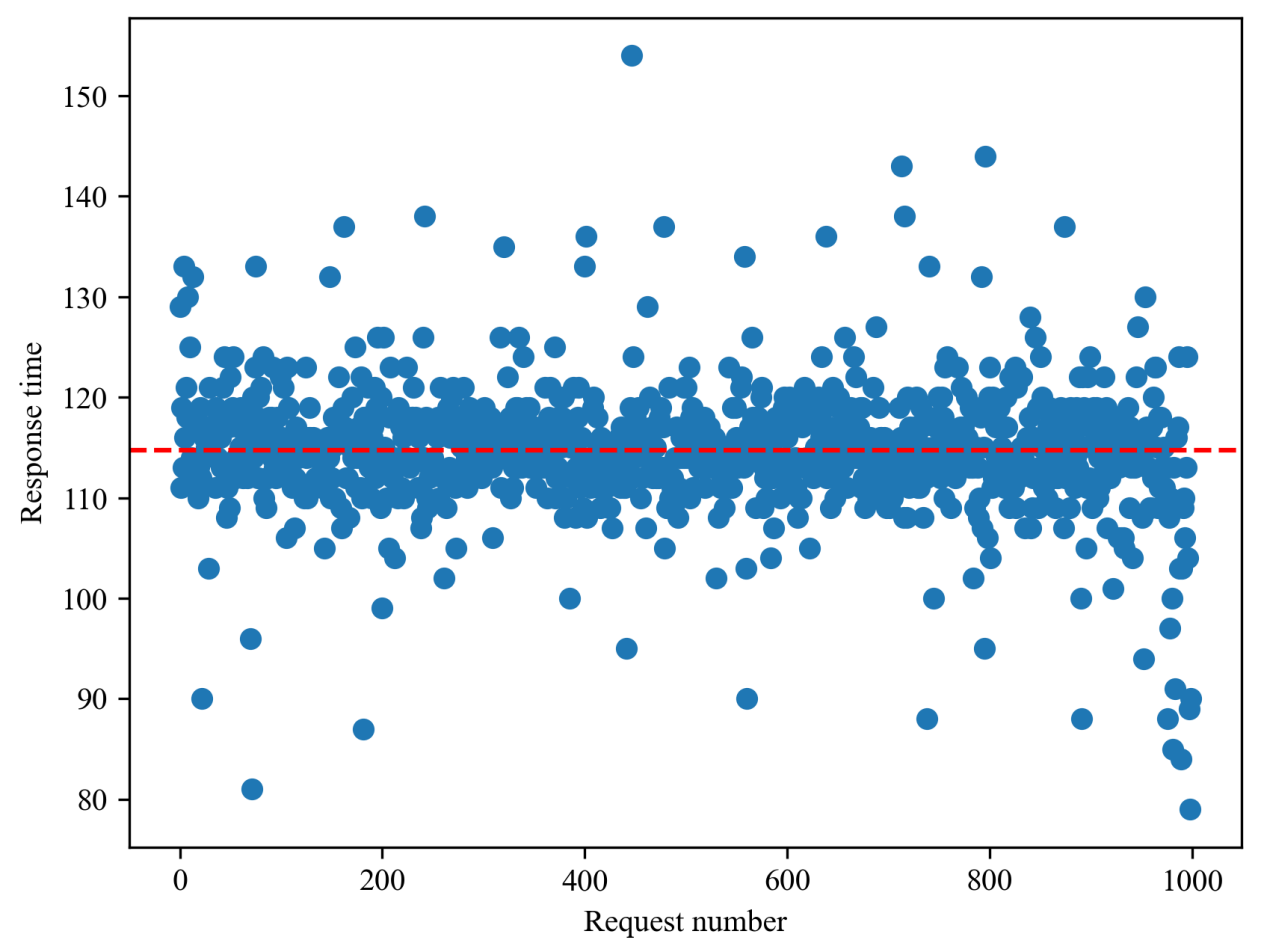
Response times for 1000 requests using random keywords.

Figure 3 provides the user interface based on Bing news articles (http://www.epidemic.co.kr/worldwide/news (accessed on 6 July 2021)). It consists of seven components. Figure 3A is the input form that allows the user to select the continent and country. Users can select one of the continents and one country. For instance, in Figure 3, Asia and China are selected as an example. When the selection is completed, the information of the corresponding country is displayed. Figure 3B presents the disease-related topic ranking based on the news articles collected with the disease keyword. It also provides the list of titles and links of contents for news articles containing the topic when it is clicked. It is updated daily, and COVID-19 is currently at the top. Since displaying topic keywords and contents is one of the main functions of EagleEye, we conducted a response time test to check user convenience. We checked response times for 1000 requests using random keywords. Figure 4 shows the distribution of response times for 1000 requests. The red dotted line drawn horizontally means the average of response times, which was 114 ms, and the maximum response time was 155 ms. Figure 3C displays a map of the country. Markers are displayed on the map, and they are the regions that international airports are located in. Users can check the number of disease related news articles of the selected district by clicking a marker. The map is updated monthly. Figure 3D shows the raking of the top five districts where the largest number of disease-related news articles is reported, and it presents the number of disease-related news articles in each district. Figure 3E shows the ranking of the top five diseases most frequently mentioned in disease-related news articles of the selected nation. The images shown in Figure 3D,E are updated monthly. Figure 3F presents the trend of this year for a monthly total number of collected news articles and a monthly number of news articles of the most frequently shown disease. Finally, Figure 3G displays the disease-related word cloud based on collected disease-related news articles. The larger the word size, the more often the word appears in the news articles. Users can select the date for all data including past data as well as the current month. The tweets user interface (http://www.epidemic.co.kr/worldwide/twitter (accessed on 6 July 2021)), includes similar components to the user interface based on Bing news.

Furthermore, our system provides data based on Google search (http://www.epidemic.co.kr/worldwide/trend (accessed on 6 July 2021)) and comparison of disease-related data obtained from the Bing News articles, Twitter tweets and Google search queries (http://www.epidemic.co.kr/worldwide/comparison (accessed on 6 July 2021)).

## 5. Experiment

We performed extensive experiments to evaluate the performance of our proposed deep-learning-based ranking algorithm (i.e., WBiLSTM-TF-IDF) and the overall system (i.e., EagleEye). The experiment consists of two perspectives; the algorithm perspective and the system perspective.

We evaluate the performance of our proposed ranking algorithm, WBiLSTM-TF-IDF, in terms of accuracy and event detection capability. For the experiment, we trained our WBiLSTM sentence classification model using 2500 pieces of articles and tweet for each news and tweets, 80% of which were training data and 20% of which were used as validation data. The training data consisted of 2000 balanced data containing half of the positive data containing disease-related information and half of negative data such as advertisements and distorted word use. In addition, we validated the trained model using remaining 500 data sets. The layer size of the model was 256, the training epochs were 100, and the input sequence length was 50.

To evaluate the accuracy of EagleEye, we compared WBiLSTM-TF-IDF with the usual TF-IDF. We used news articles and tweets collected by our system from six major countries. The news and tweets contain important data regarding actual disease outbreaks but often contain unnecessary tokens such as drug advertisements and twisted uses of words. We prepared two datasets: a clean dataset whose unnecessary data were filtered out by Word2Vec-BiLSTM and an uncleaned dataset that contains the unnecessary tokens. The tokens from the two datasets were refined and ranked by TF-IDF, and the accuracy was evaluated by selecting the top 10 words and analyzing how many words among the 10 words provided accurate information. Table 1 shows the details of the data used in this experiment.

**Figure 5:**
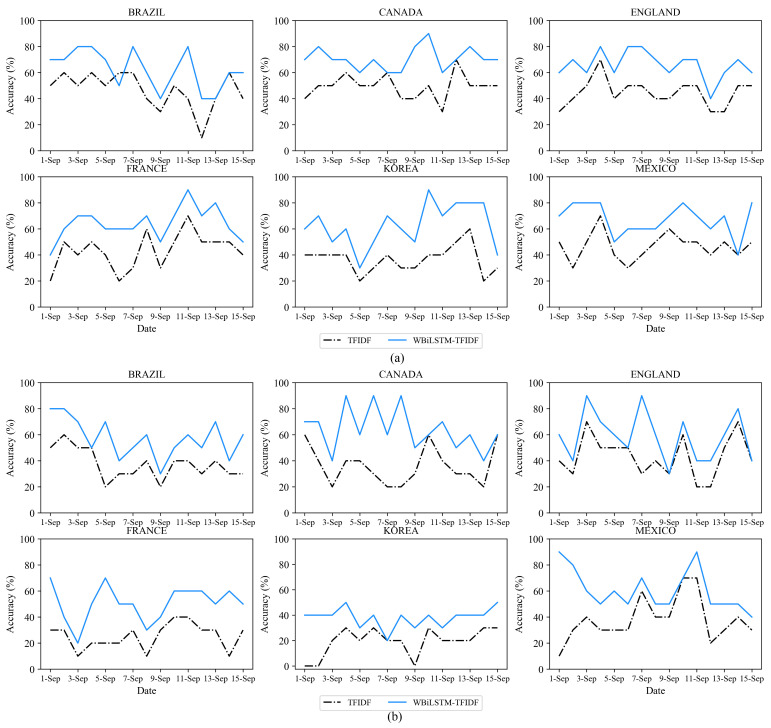
Accuracy of WBiLSTM-TF-IDF and TF-IDF as a function of the time for (**a**) news articles and (**b**) Twitter tweets.

Figure 5 shows the accuracy of WBiLSTM-TF-IDF against a typical TF-IDF as a function of time for the six major countries for news articles and tweets. In data sources and in all countries, WBiLSTM-TF-IDF surpassed TF-IDF with the highest accuracy. For news articles, the average accuracy of the six countries was 45% for TF-IDF and 65% for WBiLSTM-TF-IDF, proving that our proposed model outperforms the typical TF-IDF by 44%. For tweets, the average accuracy was 39% for TF-IDF and 55% for WBiLSTM-TF-IDF, which shows that the proposed model improves performance by 40% compared to TF-IDF. Table 2 shows the average accuracy of WBiLSTM-TF-IDF and TF-IDF.

To evaluate the event detection capability of WBiLSTM-TF-IDF, we compared the performance of WBiLSTM-TF-IDF with various existing ranking algorithms such as CCA, TF, TF-IDF, TF-IDF, Log, SMART and INQUERY. We used both news article data and Twitter tweet data collected by our system. We considered the COVID-19 event, one of the most important disease-related topics around the world in 2020, at three nations, China from 15–31 July, France from 20 September–1 October and Brazil from 1–15 July 2020. Table 3 shows the details of the data used in the experiment. Table 4 shows the ranking of disease-related events for the WBiLSTM-TF-IDF and existing ranking algorithms. For news data, WBiLSTM-TF-IDF performed better in all countries. Next, TF and SMART algorithms performed well, and CCA ranked the lowest. In the case of tweets, the proposed model showed the highest performance, followed by INQUERY. Experimental results show that our proposed model provides better event detection capability than other existing ranking algorithms.

**Figure 6:**
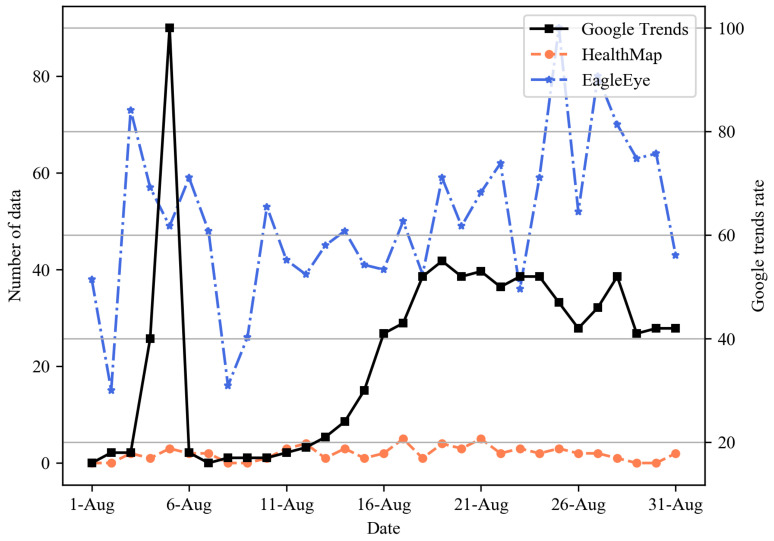
Daily time-series number of COVID-19-related data for Korea.

**Figure 7:**
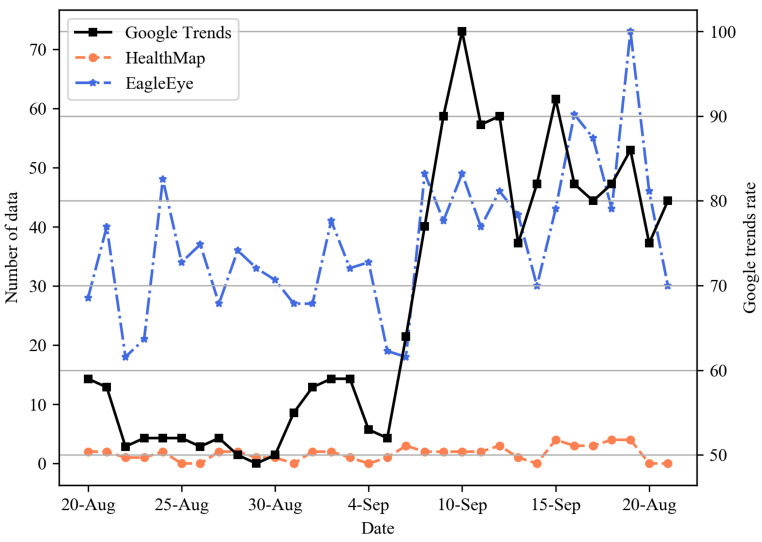
Daily time-series number of COVID-19-related data for England.

**Figure 8:**
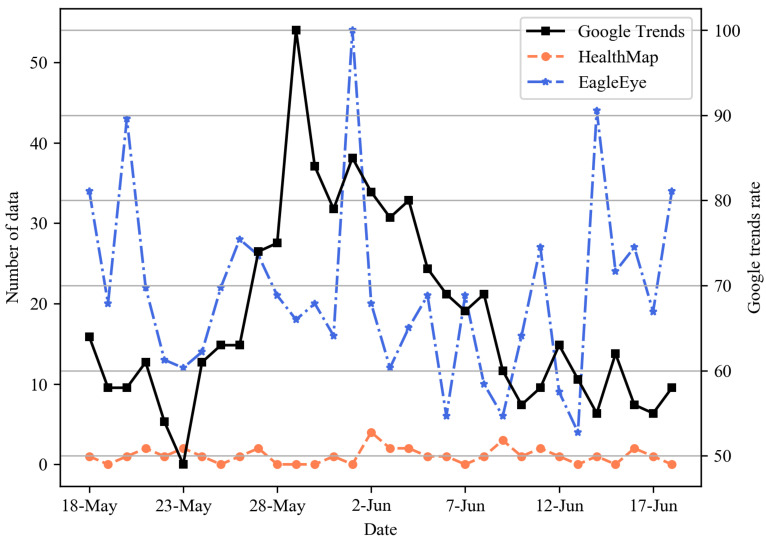
Daily time-series number of COVID-19-related data for Egypt.

To evaluate the performance of our system, we compared EagleEye with HealthMap [11], one of the representative disease surveillance systems, in terms of the event detection capability. The experiment compared how well COVID-19, which prevailed in the world, was caught in EagleEye and HealthMap in terms of the speed and the quantity. We considered three major countries: Korea from 1 to 31 August, England from 20 August to 20 September, and Egypt from 18 May to 18 June. Figure 6, Figure 7 and Figure 8 present the daily time-series number of COVID-19-related data for three nations. As a ground truth reference line for performance comparison, we added the rate of COVID-19-related Google search query data collected from Google Trends. For Korea, as shown in Figure 6, Google Trends had the largest data on 5 August, and the data flow increased as of 14 August. In EagleEye, the data flow peaked on 3 August and an increase pattern like Google Trends. On the other hand, both the number and the flow of data did not change significantly and showed a constant level in Healthmap. For England, as shown in Figure 7, the Google Trends data increased after 4 September. The data flow of EagleEye showed an increasing pattern like Google Trends. Both the number and the flow of data showed a constant level in Healthmap. Finally, for Egypt, as shown in Figure 8, Google Trends, EagleEye and Healthmap had the most data on 30 May, 1 June and 2 June, respectively, but the number of data of Healthmap was significantly smaller than Google Trends and EagleEye. Experimental results show that EagleEye responds better than HealthMap when a disease-related event occurs: EagleEye can catch disease-related events faster and gather more relevant data than the HealthMap system.

## 6. Conclusions

In this paper, we developed a novel worldwide disease-related information extraction system using various Internet-sourced data. To that end, we proposed and implemented an effective deep learning based ranking algorithm, which extracts a list of important disease-related words from Internet-sourced data combining Word2Vec, BiLSTM and TF-IDF algorithms. Through this system, people can identify important disease-related topics for countries of interest around the world and see disease-related statistics through maps, charts, correlation coefficients and word clouds. Experimental results show that our ranking algorithm is faster and more efficient in extracting disease-related subjects than existing algorithms. Moreover, the system is more sensitive to disease-related events, and hence it can collect more relevant data than conventional systems. As COVID-19 spreads around the world, international health agencies and health authorities are working hard to identify trends and prevent the spread of infectious diseases. In this context, this system not only helps to improve public health around the world but also serves as an excellent research resource for researchers. Our system remains a future research task to expand the scope of the country.

## Figures and Tables

**Table 1:** Data used for the accuracy experiment of WBiLSTM-TF-IDF.

Data Set	News	SNS
Total Data	12,309	170,029
Total Terms	394,133	3,316,276
Total Bytes	2,476,011	22,255,812
Period	1 September 2020–15 September 2020	
Nation	6 (Brazil, Canada, England, France, Korea, Mexico)	

**Table 2:** Average accuracies of WBiLSTM-TF-IDF and TF-IDF.

	TF-IDF		WBiLSTM-TF-IDF	
	News	SNS	News	SNS
Brazil	47%	45%	61%	56%
Canada	50%	40%	70%	66%
England	44%	43%	65%	58%
France	42%	38%	65%	52%
Korea	39%	19%	66%	38%
Mexico	44%	40%	64%	66%
Average	45%	39%	65%	55%

**Table 3:** Data used for the event detection capability experiment of WBiLSTM-TF-IDF.

	China		France		Brazil	
	News	SNS	News	SNS	News	SNS
Total Data	4301	1972	1953	1736	2325	329
Total Terms	132,704	26,936	9720	12,116	8647	8259
Period	15 July 2020–31 July 2020		20 September 2020–1 October 2020		1 July 2020–15 July 2020	

**Table 4:** Ranking of disease-related events.

	China		France		Brazil	
	News	SNS	News	SNS	News	SNS
TF	2.5	3.8	2	76	3.2	218
TF-IDF	2.3	7.5	3	35	6	119
SMART	2	3.6	2	76	7	90
INQUERY	5	5	5	34	8	47
CCA	20	30	20	126	179	147
WBiLSTM-TF-IDF	1.5	3	1	28	3	57

## Data Availability

Data was obtained from Twitter and Bing News. Restrictions apply to the availability of these data.
